# Validity of using multiple imputation for "unknown" stage at diagnosis in population-based cancer registry data

**DOI:** 10.1371/journal.pone.0180033

**Published:** 2017-06-27

**Authors:** Qingwei Luo, Sam Egger, Xue Qin Yu, David P. Smith, Dianne L. O’Connell

**Affiliations:** 1 Cancer Research Division, Cancer Council NSW, Sydney, Australia; 2 Sydney School of Public Health, University of Sydney, Sydney, Australia; 3 Menzies Health Institute Queensland, Griffith University, Gold Coast, Queensland, Australia; 4 School of Medicine and Public Health, University of Newcastle, Newcastle, Australia; UNITED STATES

## Abstract

**Background:**

The multiple imputation approach to missing data has been validated by a number of simulation studies by artificially inducing missingness on fully observed stage data under a pre-specified missing data mechanism. However, the validity of multiple imputation has not yet been assessed using real data. The objective of this study was to assess the validity of using multiple imputation for “unknown” prostate cancer stage recorded in the New South Wales Cancer Registry (NSWCR) in real-world conditions.

**Methods:**

Data from the population-based cohort study NSW Prostate Cancer Care and Outcomes Study (PCOS) were linked to 2000–2002 NSWCR data. For cases with “unknown” NSWCR stage, PCOS-stage was extracted from clinical notes. Logistic regression was used to evaluate the missing at random assumption adjusted for variables from two imputation models: a basic model including NSWCR variables only and an enhanced model including the same NSWCR variables together with PCOS primary treatment. Cox regression was used to evaluate the performance of MI.

**Results:**

Of the 1864 prostate cancer cases 32.7% were recorded as having “unknown” NSWCR stage. The missing at random assumption was satisfied when the logistic regression included the variables included in the enhanced model, but not those in the basic model only. The Cox models using data with imputed stage from either imputation model provided generally similar estimated hazard ratios but with wider confidence intervals compared with those derived from analysis of the data with PCOS-stage. However, the complete-case analysis of the data provided a considerably higher estimated hazard ratio for the low socio-economic status group and rural areas in comparison with those obtained from all other datasets.

**Conclusions:**

Using MI to deal with “unknown” stage data recorded in a population-based cancer registry appears to provide valid estimates. We would recommend a cautious approach to the use of this method elsewhere.

## Introduction

How to deal with missing data is a common challenge in medical and epidemiological research. There are many statistical methods which can be used to handle missing data, but some methods can lead to biased estimates. For example, complete-case analysis is the default option in most statistical software, and this has been used commonly to handle missing data by excluding cases with missing values. The risk of bias due to missing data depends on the statistical method used and the missingness mechanism, which is often broadly classified into the following three categories [1]: data missing completely at random (MCAR), data missing at random (MAR), and data missing not at random (MNAR). If data are MCAR the missingness does not depend on the response variables, neither observed values nor the missing values themselves. When data are MCAR a complete-case analysis can provide unbiased estimates. If, however, a large proportion of the data are missing, complete-case analysis will lead to a considerable loss in statistical power. If data are MAR, missingness can be explained by the observed values and does not depend on the missing values themselves, and in this situation a complete-case analysis can be biased. If data are MNAR the missingness depends on the missing values themselves, even after the observed data are taken into account.

Multiple imputation (MI) is a relatively flexible and increasingly popular approach to dealing with missing data [2, 3]. The aim of MI is to provide unbiased estimates and valid standard errors from the analysis of data that are MAR or MCAR, and it is implemented in several commonly used statistical packages. The basis of MI is explained in the published literature [4, 5]. In brief, MI replaces the missing data with ‘plausible’ values multiple times (i.e. *m* times) using an imputation model appropriate for the observed data. The validity of results obtained from the analysis of data generated by MI depends on the imputation model being approximately correct and appropriate under a MAR assumption. This imputation process will generate *m* complete datasets, each of which can be used to perform the standard analysis of interest. The analysis of choice (e.g. survival analysis) is conducted separately for each imputed data set as if they were the real complete dataset. The analysis results in *m* sets of estimates which are then combined to produce one mean estimate and standard error using Rubin’s rules, while taking into account the variability in estimates between the imputed datasets, thus reflecting the uncertainty due to the missingness [4, 5].

Stage of disease information recorded in population-based cancer registries is useful for monitoring variations in survival trends and disease progression, estimating health service demands, and evaluating or modelling the effectiveness of programs for early detection [6–9]. The New South Wales (NSW) Cancer Registry (NSWCR) is the first Australian population-based cancer registry to routinely record summary stage of disease, doing so since its inception in 1972. For prostate cancer, however, it has been shown that a large proportion (41.8% in 1999–2007) of cases are recorded as “unknown” stage in the NSWCR [6]. Previous studies have provided evidence that data with “unknown” stage for prostate cancer are not MCAR [6, 9, 10], so that when analysing these data the use of inappropriate strategies such as complete-case analysis could significantly reduce the ability to statistically control for the effect of stage on patients’ outcomes [11] and may produce biased estimates [6]. As there is a great need to use this valuable historical cancer registry data, even with a large proportion of cases with “unknown” stage, an appropriate method to handle the incomplete data for stage is required.

The MI approach to missing data has been validated by a number of simulation studies by artificially inducing missingness on fully observed stage data under a pre-specified missing data mechanism [12, 13]. These studies have generally found that MI is potentially a practical and convenient method to allow for less biased assessment of patients’ outcomes when using incomplete data on stage. However, the validity of the method when using real data with real-world missing data mechanisms, has not yet been confirmed because the “true” values of the missing data are generally unknowable. It is important to note, however, that a classification of ‘‘unknown” stage in a population-based cancer registry does not imply that clinical stage was not determined or used by clinicians to inform treatment decisions at the time of diagnosis. Therefore, for cases with “unknown” stage recorded in the cancer registry, information on stage may be available from sources other than the registry.

The NSW Prostate Cancer Care and Outcomes Study (PCOS) is a population-based cohort study that collected clinical data for men registered in the NSWCR who were aged less than 70 years and diagnosed with prostate cancer in 2000–2002 [14]. Record linkage between PCOS and NSWCR was undertaken after recruitment was completed. Men in this study who were recorded as “unknown” stage in the NSWCR were assigned a known stage in PCOS, which was considered to represent the “true” stage. The availability of the “true” stage for cases with “unknown” stage in the NSWCR enabled us to formally validate the use of MI and assess the MAR assumption. The aim of this study was to assess the validity of the use of MI for dealing with “unknown” prostate cancer stage recorded in the NSWCR using the PCOS-NSWCR linked data.

## Materials and methods

This was a population-based retrospective cohort study which was approved by the NSW Population and Health Services Research Ethics Committee (formerly known as the NSW Department of Health Ethics Committee), Cancer Institute NSW and Cancer Council NSW in April 2006 (Project No: 2005-11-017).

### Data sources

**Fig 1:**
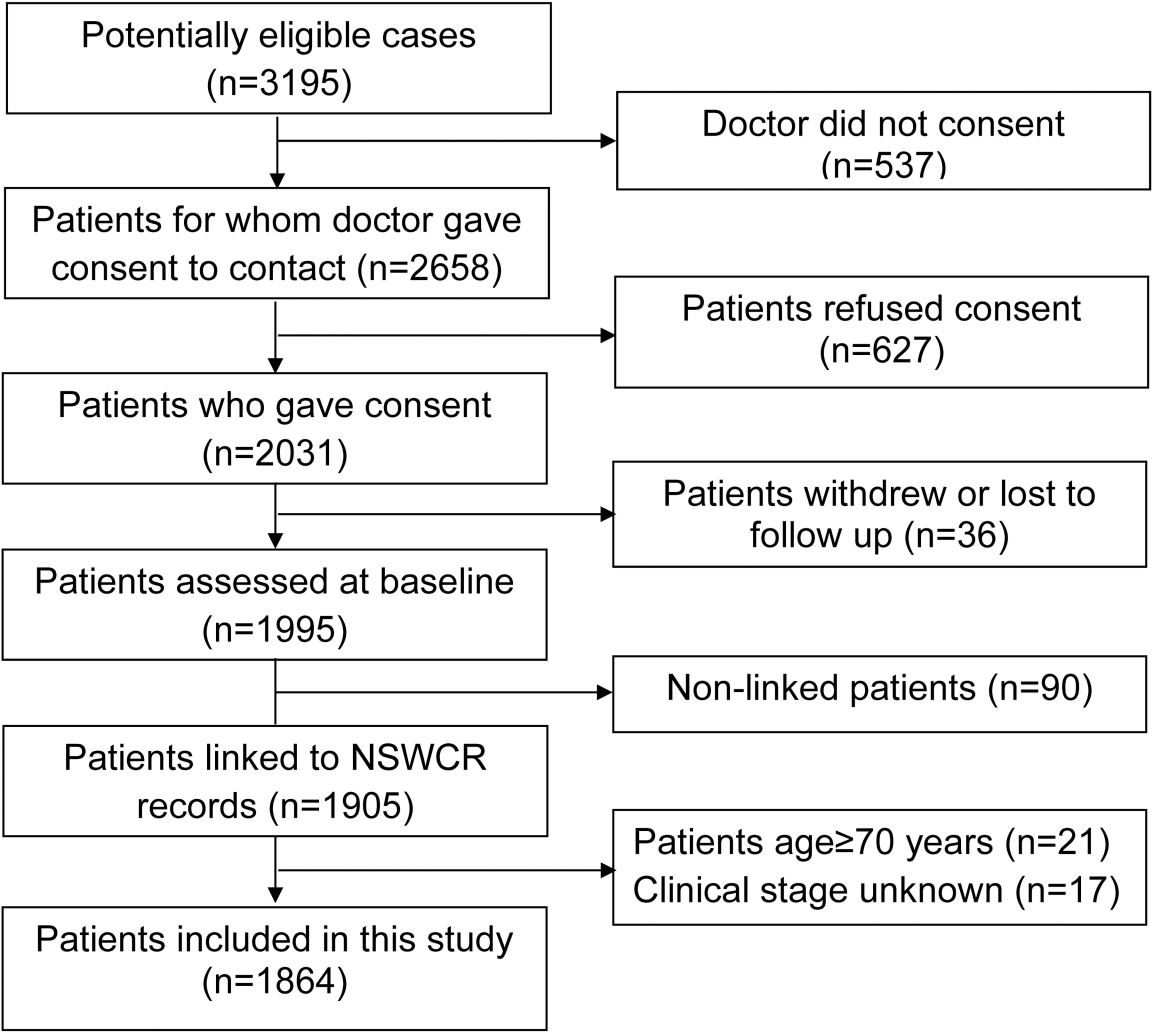
Inclusion and exclusion of prostate cancer patients in NSW Prostate Cancer Care and Outcomes Study, Australia.

Records in the population-based cohort study (PCOS) were linked with the NSWCR records by the Centre for Health Record Linkage (CHeReL). Eligible men were aged less than 70 years at first diagnosis of histopathologically confirmed prostate cancer in NSW between October 2000 and October 2002. Clinical data were extracted from patients’ medical records by trained field officers or by treating physicians using a data collection protocol [14, 15]. The stepwise inclusion and exclusion of patients for analysis is illustrated in Fig 1. After excluding 17 cases who had missing clinical stage information, 1864 prostate cancer cases were included in the current analysis.

### Stage at diagnosis from two sources of data

#### NSWCR stage

Summary stage at diagnosis as recorded by the NSWCR was based on statutory notification forms and pathology reports, and is hereafter referred to as “NSWCR stage”. It is classified using a modified summary classification [16], similar to that used by SEER [17], with spread defined as localised (cancer contained entirely in the prostate gland), regional (cancer extended into tissues surrounding the prostate or to regional lymph nodes), distant (cancer extended beyond regional lymph nodes, to bones or to other distant sites) and “unknown” (where information in the notifications was insufficient to assign stage). For some analyses, stage at diagnosis was grouped into a dichotomous variable indicating “unknown” or “known” NSWCR stage. For the purpose of MI, “unknown” NSWCR stage was coded as missing data. In this study, 32.7% of men had “unknown” NSWCR stage and this was the only variable with missing values imputed using MI.

#### PCOS-stage

For men who had “unknown” NSWCR stage, clinical stage based on tumour size (T), regional lymph nodes (N) and metastases (M) was determined from the clinical information available to the PCOS study [14]. The TNM stages were then combined to define summary stage of disease using the same classification as that used by the NSWCR. This was assumed to represent the closest approximation to the “true” stage for those cases with “unknown” stage recorded in the NSWCR, and is hereafter referred to as “PCOS-stage”.

### Other NSWCR variables

#### Survival status and survival time

Survival status and cause of death were obtained from two sources. Men with prostate cancer were matched against death records from the NSW Registry of Births, Deaths, and Marriages and the Australian Bureau of Statistics Cause of Death records. All eligible cases were followed up to the end of 2007 (the most recent data available at the time). Survival time was calculated from the date of prostate cancer diagnosis to the date of death from prostate cancer. Those who did not die from prostate cancer were censored at the date of death from other causes or at 31 December 2007 if they were still alive.

#### Demographic variables

Demographic variables that were available from the NSWCR and were used in these analyses include age at diagnosis, geographical location and socio-economic status of local government area of residence at diagnosis. Geographical location of residence was categorised into major cities, inner regional, and rural (including outer regional, remote and very remote areas) using the Australian Standard Geographic Classification Remoteness Structure [18]. This Remoteness Structure is recognised as a nationally consistent measure of geographic remoteness, based on the physical road distance to the nearest town or service centre. The Index of Economic Resources, derived from the 2001 Census, was used as a measure of area-level socio-economic status [19].

#### Primary treatment according to PCOS

Primary treatment was defined as the treatment received up to six months after diagnosis [14] and was categorised into four groups [20]: radical prostatectomy (RP), androgen deprivation therapy (ADT) or orchidectomy (OT), external beam radiotherapy (EBRT) or brachytherapy, and other treatments including active surveillance (AS) and watchful waiting (WW).

### Statistical analysis

**Fig 2:**
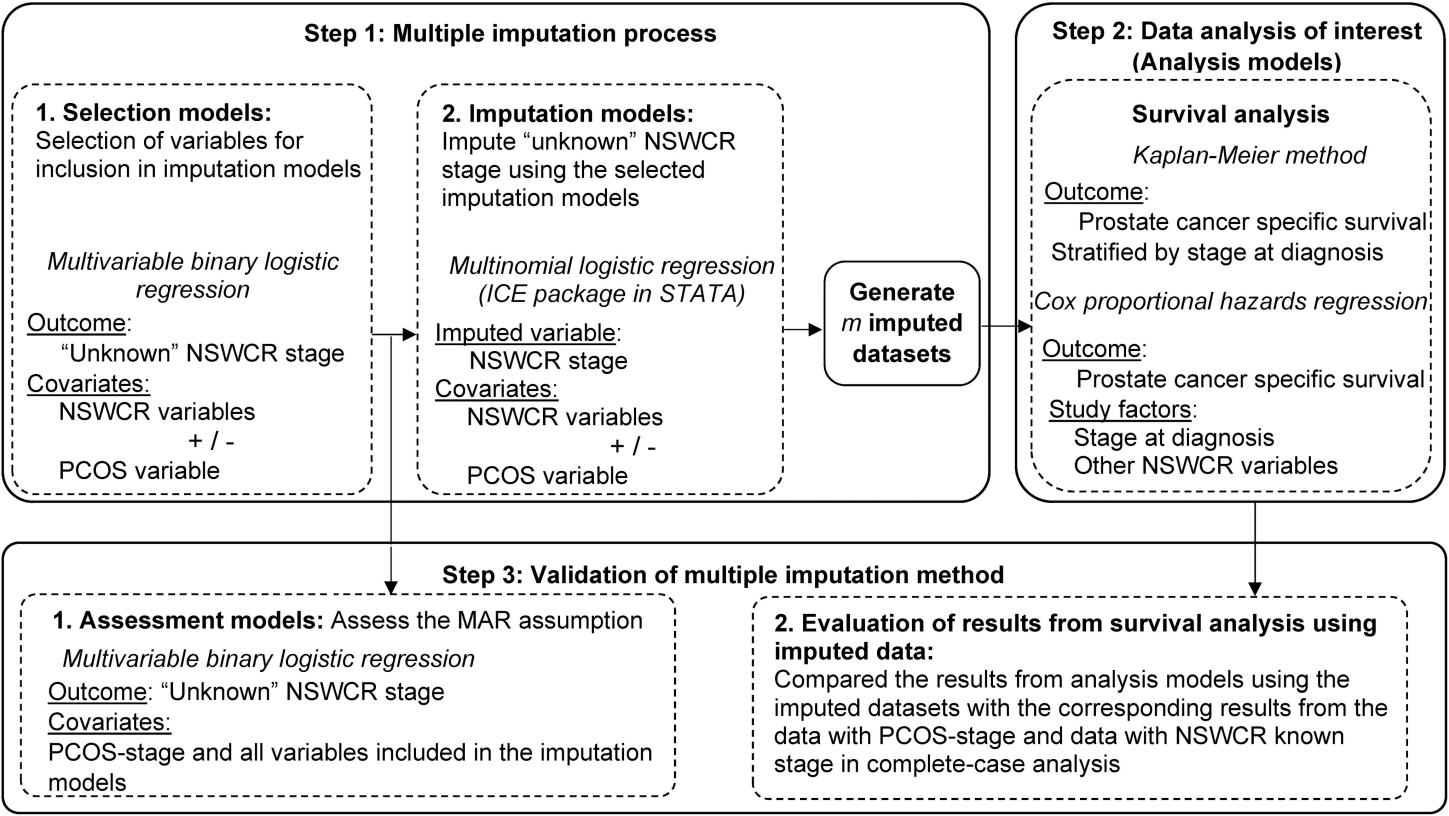
Statistical analyses included in this study. * NSWCR, NSW Cancer Registry; PCOS, Prostate Cancer Care and Outcomes Study.

There were three steps of analyses involved in this study, which are summarised in Fig 2 and described in detail below. In brief, the first step focussed on investigating the missing data mechanism and selecting variables for imputation. This step involved the selection of variables for inclusion in imputation models for “unknown” NSWCR stage (the statistical models in this step are hereafter referred to as “selection models”), and imputing “unknown” NSWCR stage using the selected models (hereafter referred to as “imputation models”). This first step generated 35 imputed datasets which were then used in the second step. The second step involved performing the data analysis of interest using the 35 imputed datasets generated in the first step. The statistical models in the second step are hereafter referred to as “analysis models”. The third step consisted of two parts: assessing the MAR assumption for “unknown” NSWCR stage and the PCOS-stage adjusting for variables included in the two imputations models (the statistical models in this step are hereafter referred to as “assessment models”). We evaluated prostate cancer-specific survival with Kaplan-Meier estimates and Cox proportional hazards regression models, and compared these with the corresponding results from the data with PCOS-stage and NSWCR known stage in a complete-case analysis.

#### Selection of variables for inclusion in imputation models for “unknown” NSWCR stage

Two general rules were applied when selecting variables for inclusion in the imputation models. Firstly, to avoid bias in the analysis model, the imputation model must include all variables intended to be included in the analysis model, including the outcomes [2, 21, 22]. Secondly, multivariable binary logistic regression was used to identify auxiliary variables that were associated with the odds of ‘‘unknown” stage being recorded in the NSWCR but are not included in the analysis models. In this study, we intended to use the imputed data in prostate cancer-specific survival analyses, therefore survival status and either the Nelson-Aalen estimator of the cumulative hazard to the survival time, H(T) [12] or the survival time (T) [2] were included in the imputation models. The correlation between H(T) and survival time T was found to be high (0.89), indicating that for these data the use of H(T) or T as a predictor for “unknown” stage made little difference, and the results obtained based on these two variables were similar. Therefore, we have only presented the results from imputation models that included the survival time (T). Survival time and age at diagnosis were included in the imputation models as continuous variables. Auxiliary variables with *p*<0.05 in either a bivariable or multivariable model were also included in the imputation models. For the purposes of comparison, the following two imputation models were selected:

1. Basic model: Restricted to variables that were available from the NSWCR database, including all variables in the analysis model (age at diagnosis, geographical location, socio-economic status, survival status and survival time). The basic model represented the observed data that are generally available to researchers using data from population-based cancer registries.
2. Enhanced model: Included all the variables in the basic model plus primary treatment (with *p*<0.0001) obtained in the PCOS study as an auxiliary variable.

#### Impute “unknown” NSWCR stage using the imputation models

As it is often suggested that the number of imputations (*m*) should be at least equal to the percentage of incomplete cases [2, 23, 24], we used *m* = 35 for this study as 32.7% (n = 610) of the study cohort had “unknown” NSWCR stage recorded in the cancer registry and this was coded as missing data. Stage at diagnosis is an ordered categorical variable but because it did not meet the proportional odds assumption when defined as the outcome variable in ordinal logistic regression, multinomial logistic regression [13] within the *ice* package in STATA [2] was used to impute NSWCR stage. For each of the 610 cases with missing data on NSWCR stage, the imputation procedure imputed a plausible stage category 35 times. Combining with the 1254 cases with a known NSWCR stage the imputation procedures created 35 complete data sets based on each of the selected imputation models, which is hereafter referred to as the “imputed stage”.

#### Data analysis of interest–survival analysis using imputed datasets

The response of interest in the survival analyses was time to prostate cancer death, or censoring due to other causes of death or end of follow-up. The prostate cancer-specific survival *S*_*E*_(*t*) was estimated in each imputed dataset using the Kaplan-Meier method, transformed using log{−log[*S*_*E*_(*t*)]} before applying Rubin’s rules to obtain a summary estimate [25, 26], and was graphically presented to allow comparisons with the survival estimates based on PCOS-stage. Cox proportional hazards regression models were performed using the ‘*mi estimate*: *stcox*’ command in STATA to examine differences in the risk of dying from prostate cancer by patients’ demographic and clinical characteristics including the imputed stage.

#### Assess the MAR assumption

A MAR pattern is present when missingness (i.e. missing vs not-missing) is dependent on the observed values but not on the actual values of the missing observations. If missingness is dependent on the actual values of the missing observations after the observed data are taken into account then the data are said to be MNAR [1]. To evaluate the MAR assumption for “unknown” NSWCR stage we used multivariable binary logistic regression to examine the association between ‘‘unknown” NSWCR stage (vs known NSWCR stage) and the PCOS-stage (localised, regional, distant), after adjusting for the predictor variables included in the two imputation models separately. In the current analysis, the MAR assumption would be violated if, in a logistic regression model, the PCOS-stage predicted “unknown” NSWCR stage (vs known NSWCR stage) independent of other observed patient characteristics. An example of MNAR would be where metastatic cases are more likely to be recorded as “unknown” stage in the cancer registry [27] independent of other observed patient characteristics.

#### Evaluation of results from survival analysis using imputed data

To understand how a variable with imputed values will behave in statistical analyses, we compared the results from cancer-specific survival analyses using the imputed datasets with the corresponding results from the data with PCOS-stage and results from complete-case analysis using only the 1254 cases with known NSWCR stage (the default method used by most statistical software). The proportional hazards assumption was satisfied based on testing the interaction of the variables included in the regression with survival time, and visual inspection of the Schoenfeld and scaled Schoenfeld residuals [28]. We conducted sensitivity analyses by repeating the analyses of MI data obtained from the enhanced imputation model with 100 imputations. All statistical analyses were performed using STATA and *p*<0.05 was considered to denote statistical significance.

## Results

Of the 1864 eligible prostate cancer cases who participated in PCOS 84.2% had localised disease, 12.6% had regional spread and 3.3% had distant stage as recorded by PCOS (Table 1). In the NSWCR however, 610 (32.7%) of these same patients were recorded as having “unknown” stage (Table 1). The median age at diagnosis was 62 years, 69.3% of patients were resident in major cities at the time of diagnosis, and 51.8% of patients received RP. After a median of 6.2 years of follow-up (range: 0.5 to 8.0 years), 112 cases had died from prostate cancer.

**Table 1:** Characteristics of prostate cancer cases aged less than 70 years at diagnosis included in this study as recorded in PCOS, NSW (N = 1864).

	Cases		Unknown NSWCR stage	
Characteristics	n	% within total	n	% within categories
**PCOS-stage**				
Localised	1569	84.2	504	32.1
Regional	234	12.6	68	29.1
Distant	61	3.3	38	62.3
**Age at diagnosis**				
Median age (years)	62		63	
**Geographical location**				
Major cities	1291	69.3	385	29.8
Inner regional	466	25.0	189	40.6
Rural	107	5.7	36	33.6
**Socio-economic status**				
High	668	35.8	177	26.5
Middle	544	29.2	163	30.0
Low	652	35.0	270	41.4
**Survival status at the end of 2007**				
Alive	1671	89.6	538	32.2
Died from prostate cancer	112	6.0	45	40.2
Died from other causes	81	4.3	27	33.3
**Years of follow-up at the end of 2007**				
Median follow-up time (years)	6.2		6.1	
**Initial primary treatment**				
Radical prostatectomy	965	51.8	137	14.2
ADT/OT	152	8.2	96	63.2
EBRT/Brachytherapy	453	24.3	256	56.5
AS/WW	294	15.8	121	41.2
**Total**	**1864**	**100.0**	**610**	**32.7**

NSWCR: NSW Cancer Registry; PCOS: Prostate Cancer Care and Outcomes Study; ADT: androgen deprivation therapy; OT: orchidectomy; EBRT: external beam radiotherapy; AS: active surveillance; WW: watchful waiting.

### Selection of imputation models for “unknown” NSWCR stage

Table 2 presents the results from bivariable and multivariable binary logistic regression models used to identify the socio-demographic and clinical factors associated with a case having an “unknown” NSWCR stage (vs known NSWCR stage). All covariates intended for use in the analysis models that were available from the NSWCR data met the criteria to be included in the basic selection model with *p*<0.05 in bivariable or multivariable models. Survival time, age at diagnosis and socio-economic status as available in the NSWCR data were the variables significantly associated with having “unknown” NSWCR stage in both the bivariable models and the basic selection model. Geographical location was only significant in the bivariable model, and survival status was included in the imputation models since it was the outcome for the analysis models. In the enhanced imputation model, survival status and survival time available in the NSWCR data and primary treatment taken from PCOS were the variables significantly associated with having “unknown” NSWCR stage. After the imputation procedure, 35 complete data sets with imputed stage were generated using each selected imputation model.

**Table 2:** Associations between sociodemographic and clinical characteristics and “unknown” NSWCR stage prostate cancer for PCOS cases (N = 1864).

Variable	Cases	(Unknown NSWCR stage %)	Bivariable model— Unadjusted odds ratio (95% CI)				Multivariable model—adjusted odds ratio (95% CI)							
							Basic selection model^a^				Enhanced selection model^b^			
**Survival status at the end of 2007**				* *	* *	*p = 0*.*20*		* *	* *	*p = 0*.*40*		* *	* *	*p<0*.*0001*
Alive	1671	(32.2)	1.00				1.00				1.00			
Died from prostate cancer	112	(40.2)	1.41	(0.96	-2.09)		0.81	(0.47	-1.39)		0.26	(0.14	-0.48)	
Died from other causes	81	(33.3)	1.05	(0.66	-1.69)		0.66	(0.36	-1.20)		0.34	(0.17	-0.66)	
**Survival time at the end of 2007**														
One year increase			0.89	(0.82	-0.97)	*p = 0*.*007*	0.85	(0.75	-0.97)	*p = 0*.*01*	0.86	(0.75	-0.99)	*p = 0*.*03*
**Age at diagnosis**														
One year increase			1.04	(1.02	-1.06)	*p<0*.*0001*	1.04	(1.02	-1.06)	*p<0*.*0001*	1.01	(0.99	-1.03)	*p = 0*.*50*
**Geographical location**						*p = 0*.*0002*				*p = 0*.*20*				*p = 0*.*08*
Major cities	1291	(29.8)	1.00				1.00				1.00			
Inner regional	466	(40.6)	1.61	(1.29	-2.00)		1.13	(0.86	-1.48)		1.25	(0.93	-1.69)	
Rural	107	(33.6)	1.19	(0.79	-1.81)		0.76	(0.48	-1.21)		0.76	(0.46	-1.26)	
**Socio-economic status**						*p<0*.*0001*				*p = 0*.*0001*				*p = 0*.*07*
High	668	(26.5)	1.00				1.00				1.00			
Middle	544	(30.0)	1.19	(0.92	-1.53)		1.15	(0.89	-1.50)		1.07	(0.80	-1.42)	
Low	652	(41.4)	1.96	(1.55	-2.47)		1.83	(1.36	-2.45)		1.43	(1.03	-1.97)	
**Primary treatment**						*p<0*.*0001*								*p<0*.*0001*
Radical prostatectomy	965	(14.2)	1.00								1.00			
ADT/OT	152	(63.2)	10.36	(7.11	-15.09)						14.32	(9.21	-22.28)	
EBRT/Brachytherapy	453	(56.5)	7.85	(6.06	-10.18)						8.32	(6.34	-10.91)	
AS/WW	294	(41.2)	4.23	(3.15	-5.67)						4.13	(3.06	-5.58)	

NSWCR: NSW Cancer Registry; PCOS: Prostate Cancer Care and Outcomes Study; ADT: androgen deprivation therapy; OT: orchidectomy; EBRT: external beam radiotherapy; AS: active surveillance; WW: watchful waiting.

a. Basic selection model includes NSWCR variables survival status, survival time, age at diagnosis, geographical location, and socio-economic status.

b. Enhanced selection model includes all variables in the basic imputation model, plus primary treatment from PCOS.

### Assessing the MAR assumption

Table 3 shows there was a significant association between “unknown” NSWCR stage and PCOS-stage independent of variables included in the basic imputation model (*p* = 0.0001), indicating that the MAR assumption is violated if the imputation model only includes variables available in the cancer registry data. However, the corresponding association was not statistically significant when variables from the enhanced imputation model (*p* = 0.50) were included (the variables from the basic imputation model plus primary treatment as recorded in PCOS), indicating that the MAR assumption is satisfied when the observed data includes the additional information on treatment.

**Table 3:** Assessment of the missing at random assumption–the associations between “unknown” stage prostate cancer recorded in the NSWCR and PCOS-stage, after adjusting for variables included in the imputation models (n = 1864).

	Cases	(Unknown stage %)	Bivariable model -			Multivariable model—adjusted odds ratio (95% CI)					
			Unadjusted odds ratio (95% CI)			Basic imputation model^a^			Enhanced imputation model^b^		
**PCOS-stage at diagnosis**			*p<0*.*0001*			*p = 0*.*0001*			*p = 0*.*5*		
Localised	1569	(32.1)	1.00		1				1.00		
Regional	234	(29.1)	0.87	(0.64	-1.17)	0.85	(0.62	-1.15)	0.94	(0.66	-1.32)
Distant	61	(62.3)	3.49	(2.06	-5.92)	3.24	(1.80	-5.82)	1.44	(0.75	-2.76)
**Missing at random assumption**	** **	** **	Not satisfied			Not satisfied			Satisfied		

NSWCR: NSW Cancer Registry; PCOS: Prostate Cancer Care and Outcomes Study

a. Adjusted for variables from the basic imputation model including NSWCR variables: survival status, survival time, age at diagnosis, geographical location, and socio-economic status.

b. Adjusted for variables from the enhanced imputation model: all variables in the basic imputation model plus primary treatment from PCOS.

### Evaluation of results from survival analysis using imputed data

**Fig 3:**
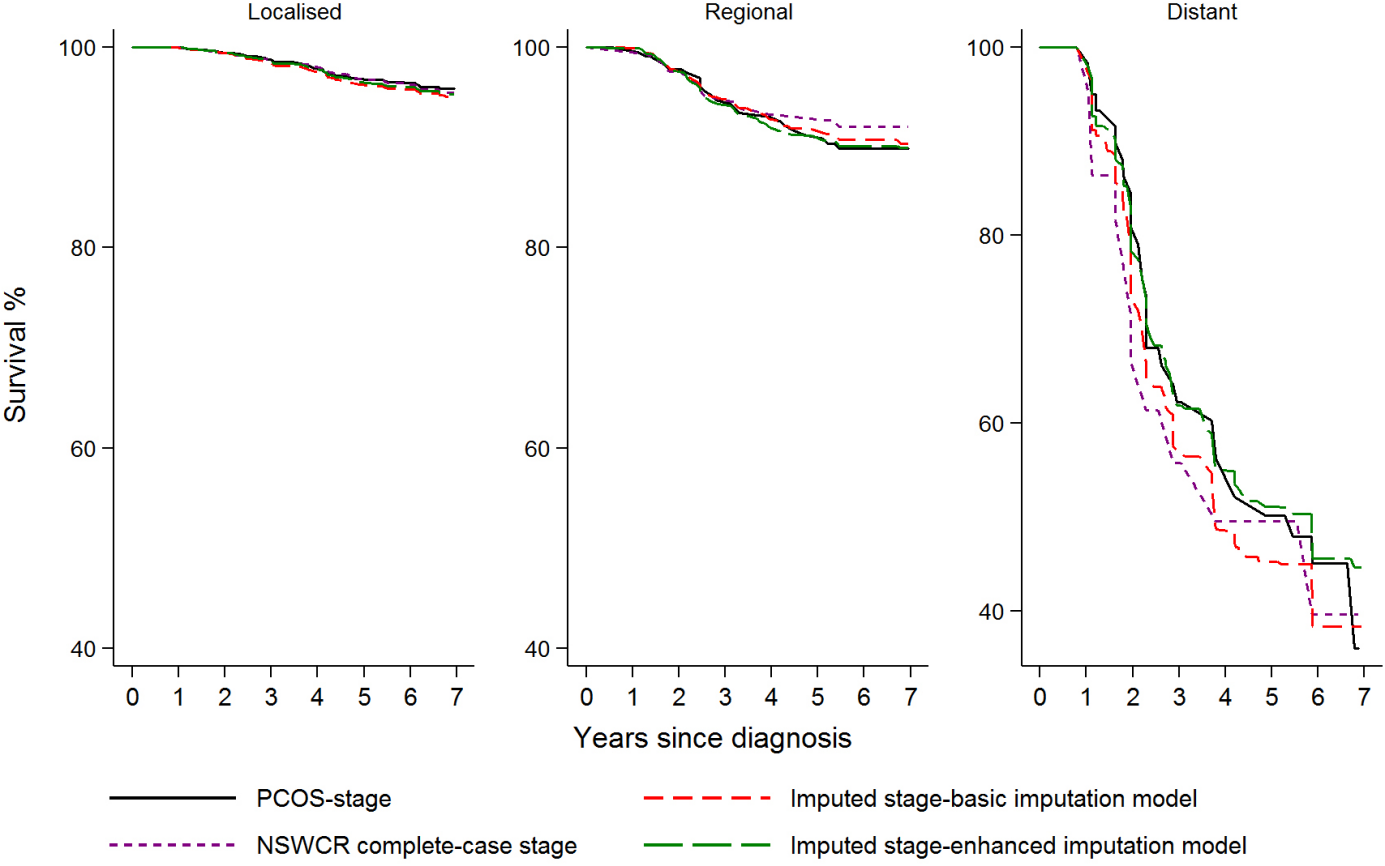
Comparison of Kaplan-Meier curves for prostate cancer-specific survival based on analyses of PCOS-stage data, the NSWCR complete-case stage data and imputed stage data from two imputation models. * NSWCR, NSW Cancer Registry; PCOS, Prostate Cancer Care and Outcomes Study.

**Fig 4:**
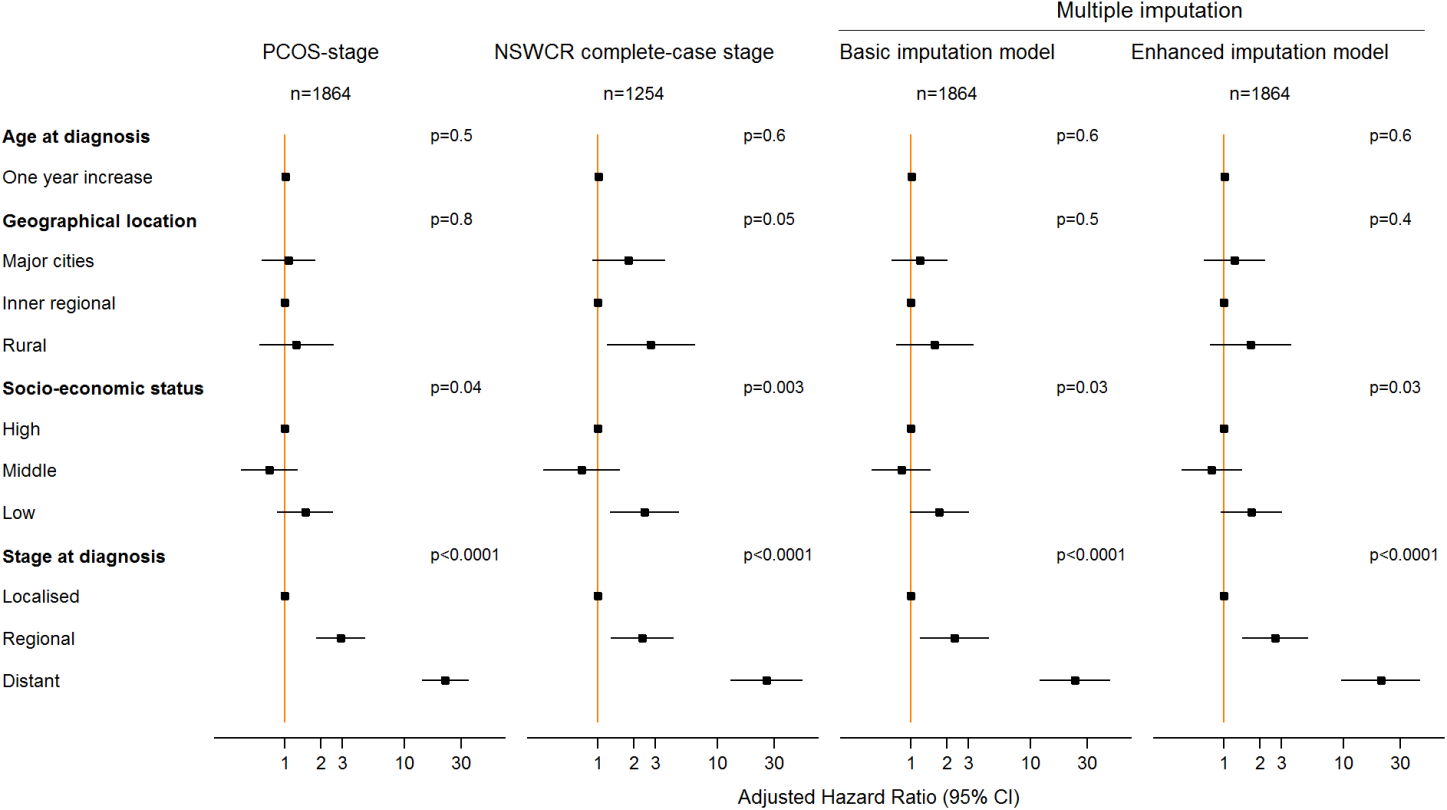
Comparison of the estimated adjusted hazard ratios from multivariable Cox proportional hazards regression models for data with PCOS-stage, the NSWCR complete-case stage and imputed stage from two imputation models. * NSWCR, NSW Cancer Registry; PCOS, Prostate Cancer Care and Outcomes Study.

Kaplan-Meier prostate cancer-specific survival estimates by stage at diagnosis for various datasets including the datasets with imputed stage are shown in Fig 3. Compared to the analysis of the dataset with PCOS-stage, analysis of the dataset derived from the basic imputation model appears to underestimate survival for patients with distant stage disease. Analysis of the dataset derived from the enhanced imputation model provided similar survival estimates compared to those obtained from the analysis of the dataset with PCOS-stage (Fig 3). In the multivariable Cox proportional hazards regression models (Fig 4 and S1 Table) examining the associations between prostate cancer-specific survival and socio-demographic factors and stage at diagnosis, analysis of data from both imputation models provided generally similar estimated hazard ratios (HRs) but wider confidence intervals (CIs) compared with those derived from analysis of the data with PCOS-stage. The complete-case analysis of the data with NSWCR stage provided similar HR estimates for stage (HR: 2.35, 95% CI: 1.28–4.31 for regional and HR: 25.71, 95% CI: 12.90–51.23 for distant stage) compared with the estimates from data with PCOS-stage (HR: 2.91, 95% CI: 1.81–4.69 for regional and HR: 22.04, 95% CI: 14.08–34.52 for distant stage). However, the complete-case analysis of the data provided a considerably higher estimated HR for the low socio-economic status group (HR: 2.45, 95% CI: 1.25–4.79) and rural areas (HR: 2.76, 95% CI: 1.18–6.46) in comparison with those obtained from all other datasets. The results from sensitivity analyses with 100 imputed datasets using the enhanced imputation model were similar to the corresponding results from 35 imputations (S1 Table).

## Discussion

To our knowledge this is the first study to develop and evaluate the MI method for “unknown” prostate cancer stage recorded in a population-based cancer registry using external clinical information on stage of disease through record linkage. We have shown that in a representative sample of men diagnosed with prostate cancer, MI was a valid method for dealing with “unknown” stage recorded in a population-based cancer registry database, and to reflect the uncertainty associated with “unknown” NSWCR stage. MI using the basic imputation model comprising only those variables available in the dataset from the NSWCR appeared to produce similar parameter estimates for all study factors included in this study, compared to the MI using the enhanced imputation model.

MI is a statistical method for handling missing data and has many advantages when correctly implemented. The aim of MI is to provide unbiased statistical estimates while accounting for the uncertainty caused by the missing data when it is MAR. Although MI is appealing and has become increasingly popular due to the ease of implementation in most common statistical software packages [2, 29], an inappropriate imputation method may provide biased estimates [1]. For researchers using MI it is important to note that the validity of the method is dependent on the missingness mechanism and several procedural requirements [2, 3, 22–25]. First, the correct regression method for the imputation model (linear regression, ordinal logistic regression or multinomial logistic regression) must be specified based on the type of variables to be imputed [2]. Second, the imputation model must include the covariates and outcome from the planned analysis model, and all variables predictive of the incomplete variable or influencing the process causing the missing data, even if these variables are not of interest in the intended analysis [2]. Third, the number of imputations should be selected based on the statistical efficiency of the estimates, or as it is often suggested that the number of imputations (*m*) should be at least equal to the percentage of cases with incomplete data [2, 23, 24]. Fourth, the imputation models must be checked for any potential perfect prediction problems when categorical variables are involved (i.e. when one or more observations has fitted probability exactly zero or one) [2]. Fifth, justification of the plausibility of the MAR assumption must be provided through consideration of all the possible reasons for missing data [2, 3], through assessing the differences between the results from complete-case analysis and MI [2, 3]. It is also suggested that sensitivity analyses to investigate the robustness of inferences to estimate the parameters under MNAR mechanisms are desirable, although this requires ongoing research [3, 30]. Last but not least, sufficient details about the imputation methods should be provided as supplements when reporting the results in the epidemiological literature [3].

For standard MI to be valid, the primary assumption that the data are MAR needs to be approximately correct, but is not a property of the data [3]. In this study, the MAR assumption was satisfied if the primary treatment received was a part of the observed data, but not if the variable was not included in the observed data (as was the case for the basic imputation model included only data available in the NSWCR). While treatment data was not available in the NSWCR data, it is not uncommon for cancer registries to collect this information. The USA SEER database, for example, has data on treatment, and the recently established NSW Prostate Clinical Cancer Registry intends to capture cancer treatment information that will be incorporated into the NSWCR. The availability of treatment data might enable researchers to conduct a valid MI under MAR.

With a considerably large proportion of missing data, the performance of MI can be sensitive to departures from the MAR assumption [2]. There have however been some developments in methods for dealing with MNAR data [31, 32]. In addition, the standard MI model can be extended to incorporate specific MNAR mechanisms by using a weighted approach or by modelling speculated mechanisms for missingness [2, 4, 30]. Nevertheless, it is common for the missing data mechanism to actually consist of both observed and unobserved factors, and the effects of an inaccessible mechanism are often minimal in the implementation of MI [33]. Hamilton and colleagues [34] reported that MI appears reasonably robust under a MNAR mechanism. This was confirmed in the current study using real data, as the MI implemented under a MNAR mechanism using the basic imputation model appeared to still provide relatively unbiased estimates. This may be because there is a strong association between stage at diagnosis and cancer survival, and the survival outcomes have been included in the imputation models. In addition, we found that cases with distant stage were more likely to be recorded as “unknown” stage in the NSWCR than cases with localised or regional disease. However, in this study only a very small number of cases (less than 5% of the study cohort) were diagnosed with distant PCOS-stage, which may be another reason why the departure from a MAR assumption had minimal effects on the analysis models using imputed data.

Without knowing the mechanism leading to the missing data, it is difficult to interpret comparisons of estimates from the analysis of imputed data with those from the complete-case analysis. If the missing data are confirmed to be not MCAR, the estimates from analyses of the imputed data may differ from those from complete-case analysis, particularly if the missingness is associated with both the study outcome of interest and other study factors. We found that the complete-case analysis of the NSWCR dataset in this study provided similar estimates for stage compared to the estimates using data with PCOS-stage and those from the enhanced imputation model. However, the complete-case analysis appeared to overstate the risk of dying from prostate cancer for cases from rural areas or areas with low socio-economic status. A possible reason for this might be due to the differences between cancer services in different socio-economic areas or geographical locations, and insufficient recording of clinical data affecting the NSWCR’s ability to determine stage. The large proportion of cases with “unknown” NSWCR stage may have resulted in residual confounding by stage. The findings from this study provide a good example of the potential for differences between estimates from the analysis of MI data and those from complete-case analysis for a variable other than the imputed variable. This demonstrates by way of example that comparisons of the results obtained from complete-case analysis and those obtained from MI should not be used to evaluate the performance of MI.

Consistent with two simulation studies of MI on stage of disease for male colorectal cancer, melanoma and female breast cancer [12, 13], this validation study using real data confirmed that MI using multinomial regression may, under certain conditions, be an appropriate method for dealing with “unknown” prostate cancer stage in a population-wide cancer registry. Our analysis also suggests that complete-case analysis which excludes prostate cancer cases with “unknown” stage can produce biased estimates. Another population-based study [35] reported a tutorial on MI using ordinal regression models for colorectal cancer stage under the assumption that the missing data are MAR. However, the results were not directly validated because, like most analysis, the “true” stage was not available in that study. Another study of the validity of MI for variables other than cancer stage which used linked ambulance records [36] reported fair to good agreement between the imputed values for variables in a state trauma registry when compared with known values from an additional data source, although this study unfortunately did not report a formal statistical test of the MAR assumption as presented in our study.

The main strength of our study is that, unlike the many previous simulation studies of MI, it has demonstrated the potential benefits of MI in a real-world setting using a state-wide population-based cohort study and record linkage. The study cohort did not differ much in age, area of residence or socio-economic status from all patients aged less than 70 years at diagnosis with prostate cancer registered during the recruitment period [14]. Also, the overall survival pattern by NSWCR stage at diagnosis in PCOS is consistent with that for the whole NSW population of prostate cancer cases aged less than 70 years at diagnosis in 2000–2002 (S1 Fig). This study is limited to prostate cancer in NSW, so findings might not be generalisable to other cancer types or other jurisdictions without reasonable justification. Also another limitation is the potential differential misclassification due to the differences in cancer stage recorded in the two data sources, although of the cases with known disease stage recorded in both PCOS and the NSWCR the agreement was 82%.

In conclusion, we found that while MI should be used with care and the results interpreted with caution, MI for data on stage recorded in a population-based cancer registry appears to provide valid estimates when the method was correctly implemented. Using real data we demonstrated that in some situations standard MI under the MNAR assumption may be valid when the effects of an inaccessible missing mechanism are minimal, but MI under MNAR should be used with caution as it is sensitive to the degree of departure from the MAR assumption [2]. Historical cancer registry data are of great value to researchers, so it is important that appropriate methods for dealing with unavoidable missing data are investigated and validated. We hope that our method of validation of the MI method using population-based health data linked with external data sources may be applicable elsewhere, and that it will help to improve the utilisation of data from other population-based cancer registries.

## Supporting information

S1 Fig Kaplan-Meier survival curves by NSWCR stage at diagnosis in PCOS data with the whole NSW population of prostate cancer cases aged less than 70 years and diagnosed in 2000–2002. * NSWCR, NSW Cancer Registry; PCOS, Prostate Cancer Care and Outcomes Study.
(TIF)

S1 Table Comparison of the estimated adjusted hazard ratios (HRs) from multivariable Cox proportional hazard regression models for data with PCOS-stage, NSWCR complete-case stage and imputed stage from two imputation models. (PDF)
