# Supplementary figures and images for: Validity of using multiple imputation for "unknown" stage at diagnosis in population-based cancer registry data
Source: PLoS One. 2017 Jun 27;12(6):e0180033. doi: 10.1371/journal.pone.0180033 (PMC5487067; doi:10.1371/journal.pone.0180033)

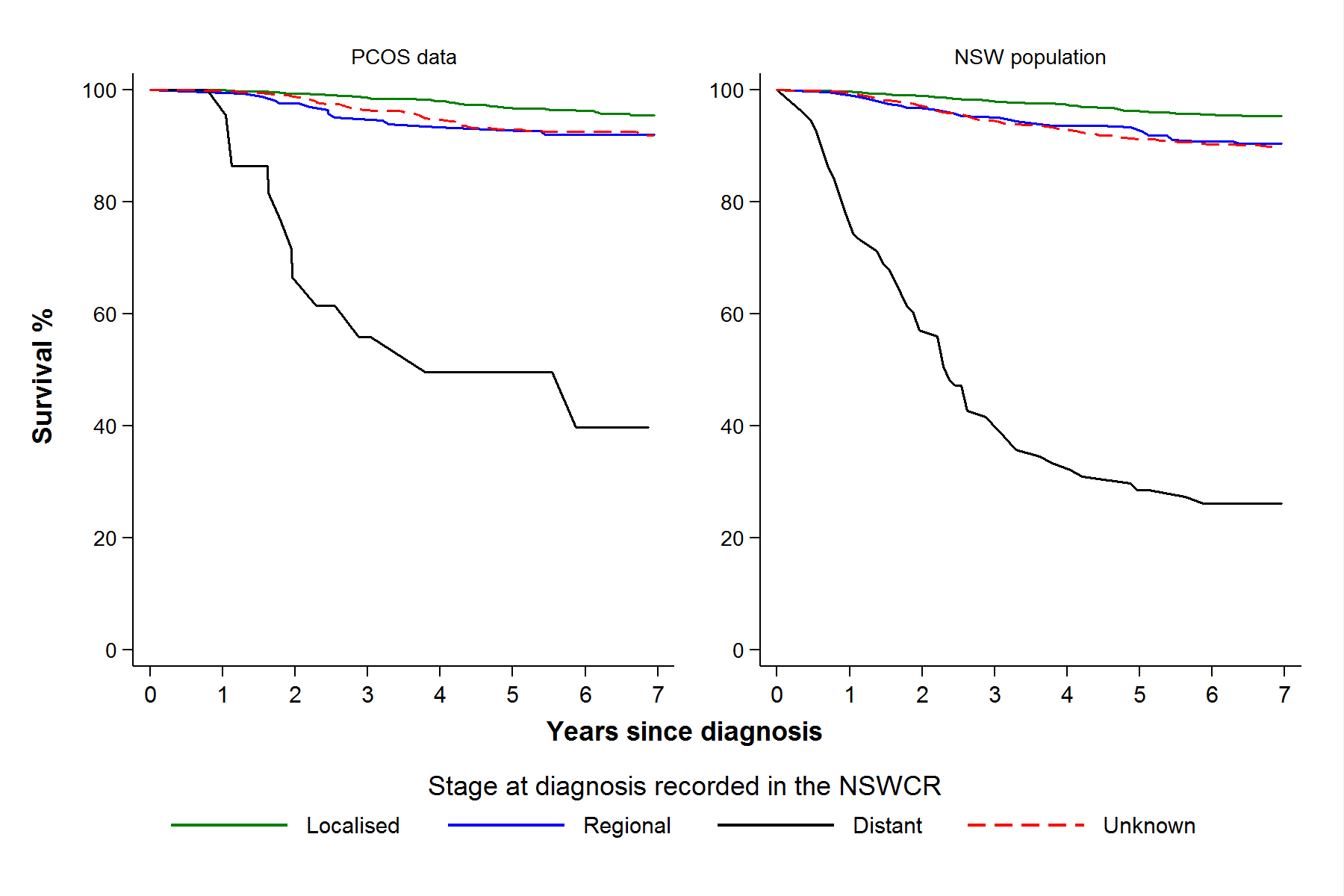

Supplement: S1 Fig — * NSWCR, NSW Cancer Registry; PCOS, Prostate Cancer Care and Outcomes Study. (TIF) [file pone.0180033.s001.tif]
