# Supplementary material for: Validity of using multiple imputation for "unknown" stage at diagnosis in population-based cancer registry data
Source: PLoS One. 2017 Jun 27;12(6):e0180033. doi: 10.1371/journal.pone.0180033 (PMC5487067; doi:10.1371/journal.pone.0180033)
Supplement: S1 Table — (PDF) [file pone.0180033.s002.pdf]

S1 Table. Comparison of the estimated adjusted hazard ratios (HRs) from multivariable Cox proportional hazard regression models for data with PCOS-stage, NSWCR complete-case stage and imputed stage from two imputation models.

	Multivariable model - adjusted hazard ratio (95% CI)									
					Data generated by multiple imputation					
	PCOS-stage		NSWCR complete-case stage		Data from basic imputation model[a]		Data from enhanced imputation model[b]		Data from enhanced imputation model[b]	
					**m=35**		**m=35**		**m=100**	
	n=1864		n=1254		n=1864		n=1864		n=1864	
**Variables**					MNAR		MAR		MAR	
**Age at diagnosis**	p=0.5		p=0.6		p=0.6		p=0.6		p=0.6	
One year increase	1.01	(0.98 -1.05)	1.01	(0.97 -1.06)	1.01	(0.97 -1.05)	1.01	(0.97 -1.05)	1.01	(0.97 -1.05)
**Geographical location**	p=0.8		p=0.05		p=0.5		p=0.4		p=0.8	
Major cities	1.07	(0.64 -1.79)	1.80	(0.89 -3.67)	1.18	(0.69 -2.02)	1.22	(0.68 -2.20)	1.24	(0.70 -2.21)
Inner regional	1.00		1.00		1.00		1.00		1.00	
Rural	1.24	(0.61 -2.55)	2.76	(1.18 -6.46)	1.57	(0.75 -3.32)	1.66	(0.76 -3.64)	1.63	(0.75 -3.56)
**Socio-economic status**	p=0.04		p=0.003		p=0.03		p=0.03		p=0.03	
High	1.00		1.00		1.00		1.00		1.00	
Middle	0.74	(0.43 -1.28)	0.73	(0.35 -1.52)	0.83	(0.47 -1.45)	0.79	(0.44 -1.41)	0.81	(0.46 -1.45)
Low	1.48	(0.86 -2.53)	2.45	(1.25 -4.79)	1.73	(0.98 -3.04)	1.69	(0.94 -3.05)	1.75	(0.97 -3.16)
**Stage at diagnosis**	p<0.0001		p<0.0001		p<0.0001		p<0.0001		p<0.0001	
Localised	1.00		1.00		1.00		1.00		1.00	
Regional	2.91	(1.81 -4.69)	2.35	(1.28 -4.31)	2.31	(1.19 -4.49)	2.67	(1.42 -5.04)	2.76	(1.48 -5.13)
Distant	22.04	(14.08 -34.52)	25.71	(12.90 -51.23)	23.54	(11.97 -46.31)	20.54	(9.55 -44.16)	20.99	(9.81 -44.92)

a. Basic imputation model includes NSWCR variables survival status, survival time, age at diagnosis, geographical location, and socio-economic status.
b. Enhanced imputation model includes all variables in the basic imputation model, plus primary treatment from PCOS.

* NSWCR: NSW Cancer Registry; PCOS: Prostate Cancer Care and Outcomes Study; MNAR: Missing not at random; MAR: Missing at random.
